# Medical News and Miscellany

**Published:** 1886-11

**Authors:** 


### MEDICAL NEWS AND MISCELLANY.

### "RESIGNATION" (S) (?)

There is a certain Faculty,—what e'er may be pretended,—
That made some vacant chairs;
There is a certain college,—extensively defended—
Yet, one dead Dean is there." (*Wrong fellow.*)

DR. W. H. WILKES, late of Waco, Texas, has, we learn, located at Kansas City, Mo., and has formed a co-partnership with Dr. Jackson. Our loss, is our neighbors gain ; Texas can ill afford to part with such men as Dr. Wilkes, and we shall miss him.

BATTEY'S OPERATION.—"Antisepsis in Ovariotomy." Dr. Battey reports seventy consecutive operations, and sixty-eight recoveries. He uses carbolized spray, carbolized silk for pedicle and sutures, and solution C. A. 1 to 40 for sponges and instruments.

### FALSE PRETENSE (?)

It was given out that what won the next State Medical Association meeting for Austin was the understanding that there should be no banquet and no wine! We learn that while *some* were so told, it was quietly, but extensively circulated that the best whiskey in the State is to be had at Austin! "Mc." says this is "the hatchet that did it," or, rather, we suspect that *he* did it with that hatchet.

### THE LATE CROP.

What amount, and for what, did varioloid?
What was it that the bone felon? Was it fractured?
Who knows what shape a diagnosis?
What was that for which a germicide?
How high did she expect to rate?—Did anyone dis-sputa?
Which of the ancients dyed his beard? Thucy-dides!

—Next.

### "TAIT IN TEXAS."

A local journal points with pride to the fact that Tait's operation has probably been performed nearer fifty, than five times in that State. At this rate the mothers of Texas will soon need another champion. *New York Medical Record.*

While we have a voice to speak, and an arm to strike, the *mothers of Texas* shall *never* want a champion to defend them against the vulgar attacks of renegade Southerners!

### AN ENTERPRISING WEEKLY.

After "He Snatched Her from the Grave," "Doctoring an African King," "Fees for Noted English Physicians," "Grafts of Frog's Skins," and a few more "chestnuts" of that sort have been going the rounds of the *Monthly Medical Journals* for the last year, they are reproduced in the November 6, 1886, number of a certain Eastern *Weekly* with a "sunset cover" (?) Here is enterprise for you, and shows a vigorous use of the scissors on old files.

### REPORT YOUR CASES.

The gradual additions to human knowledge that have been made year by year for centuries past stand now collated as the sum total of information of the people of the nineteenth century. That which was worthy, that which was calculated to redound of the benefit of the human race, has been preserved and put into permanent shape, accessible to all; that which was worthless has been forgotten. As with general, so with medical knowledge. It is the aggregation of individual experiences, sifted, sorted, reasoned upon, and elaborated, that makes up the sum of our knowledge of to-day. No case is too trivial to be instructive to the thoughtful and observant physician. In every such case a man will find something to ponder upon, and having so pondered, he should give the benefit of his reflections to his brethren.

The experiences and reflections of the *practical* man should be recorded in the medical periodicals. Then comes along the literary man, who gathers about him this mass of valuable information, sorts the chaff from the wheat, arranges, brings all this knowledge into order, and the valuable textbook is the result.

The majority of men seem afraid of the pen. There is scarcely a physician who will not readily sit down and tell you of some interesting experience that he has had, and tell it well, so that he instructs you; but ask him to put what he has said into writing for publication, and he at once says either that he is "*not much at writing*," or has no time, or puts it off for the *to-morrow* that never comes. It is almost inconceivable how much valuable material is thus annualliy consigned to oblivion. This is to be greatly regretted, and we desire to strongly urge upon all physicians to "write more." Never mind, if you do not possess a "literary style," or if your pen does not glide over the paper with the facility of a Gross, a Bartholow, a Stille, or a Flint; send in your contributions clad in good, honest *homespun*, and we will find a place for them—for it is just such plain, practical material that, in our judgment, tends to increase our knowledge of the healing art.

The above, from the editorial columns of the *Medical and Surgical Reporter*, so fully "covers the ground," and expresses our sentiments and wishes on this important subject, that we copy it entire, with thanks. We cannot too strongly impress upon physicians, and especially those who have not yet grown gray in the practice, the importance of recording their observation for the benefit of posterity, at least;—*it is a solemn duty.*

---

### THE LAW IN REGARD TO PUBLICATIONS.

I. Subscribers who do not give notice to the contrary are considered as wishing to continue their subscription.

II. Any person who takes a JOURNAL regularly from the post office, whether he has subscribed or not, is responsible for the subscription.

III. If subscribers move to other places without informing the publisher, they are legally responsible. Notices should always be given of the removal.

IV. Any person ordering his paper to be discontinued must pay up all arrearages, or the publisher may continne to send it until payment is made, and collect the whole amount, whether it is taken out of the office or not.

V. Postmasters are required to give notice by letter when a subscriber does not take his paper from the office.

---

IN our advertising pages, to-day, will be found a striking and instructive illustration of the comparative worth of the various kinds of baking powders now in the market.

---

CHOLERA has made its appearance in Beunos Ayres. The significance of this will be apparent when it is remembered, that, between this date and Christmas, eleven vessels from that port will arrive at New Orleans. Look out.

---

### AFTER THE QUACKS.

Our neighbors of the Tennessee State Medical Association, at a recent meeting appointed a committee, of which Dr. F. L. Sim, the indefatigable editor of the M. V. Med. Monthly is chairman, to urge upon the legislature the importance of enacting a law to regulate the practice in Tennessee ; and the committee are out in an address to the profession of that State, appealing to them to use their influence at home to favorably impress representatives, by explaining to them the objects desired, and the benefits to be obtained by such a law. Our neighbors, like ourselves, complain that the State is overrun by the refuse, driven out of other States, by the operation of such laws. We tender them our sympathy, and wish them better luck than the Texas Association had in a similar undertaking.

---

### YOUNG DOCTORS TAKE NOTICE.

Wanted—A physician, or a lady, in every county in Texas, to take subscriptions for THIS JOURNAL, and applications for membership in the *Physician's Mutual Benefit Association of Texas.* Commissions on each, *one half* for *the other;* and with every cash remittance of *Fifteen Dollars* a premium of $2.50 in cash additional, or a Hypodermic syringe, late style ; or a new and late standard medical work, worth from $2.50 to $5.00. Get every regular physician in the county to take the JOURNAL, and to join the Benefit Association. Write for blanks.

EDITOR DANIEL'S TEXAS MEDICAL JOURNAL.

---

☞ A double edition of the JOURNAL, with a splendid Moss-type engraving of the late lamented Secretary of the State Medical Association, Dr. W. J. BURT, will be published in April.

---

WANTED—A real good experienced canvasser for "*Biography of Contemporary Physicians of Texas*," edited by Dr. F. E. Daniel.
Address; S. H. DIXON, Austin, Texas.

---

WANTED—Situation in a drugstore as prescriptionist, by a young man aged 22½, of excellent habits, *thoroughly competent* and of seven years consecutive experience. Very best testimonials as to character, habits and capacity.

ADDRESS THIS OFFICE.

[We endorse applicant in every particular—EDITOR.]

---

PRIZE ESSAY OF THE TEXAS STATE MEDICAL ASSOCIATION.—Dr. D. R. Wallace, Superintendent State Lunatic Asylum at Terrell will review Dr. Brigg's paper in the next issue of this JOURNAL. The paper appears in the Transactions of this year.

---

### THE TRANSACTIONS T. S. M. A.

*Explanation:* A wrapper was addressed to each member, and sent to the bindery. Four hundred copies were finished on the 15th, and sent off. The next and last lot was finished and shipped on 27th. This is why some received the book earlier than others; no *preference* was shown.

---

### PROFESSIONAL SECRECY.

Dr. A. Y. P. Garnett gave a certificate of sickness to a clerk in the Pension office, who sent it in, to account for his absence. The officer demanded to know, specifically, *what was the matter* with the man; wanted to be, *himself*, the judge, we suppose, whether it were sufficient cause for absence. Of course Dr. Garnett refused to comply with the demand, and Secretary Lamar had the good sense to sustain him.

### PHYSICAN'S MUTUAL BENEFIT ASSOCIATION.

Assessment No. 3 for the benefit of the heirs of Dr. Albert Welch, has been paid by all but nine of the members, and the money has been sent by draft to Mrs. Welch at Paris. We hope to present her receipt in the next issue.

### THE NEW SURGEON-GENERAL U. S. A.

The President has appointed Assistant Medical Purveyor John Moore, to the office of Surgeon-General made vacant by the retirement of General Murray. We were much in hopes the President would see the justice of recognizing Dr. Baxter's claims to promotion, he being the Senior Assistant Surgeon-General and Chief Medical Purveyor. But then—Baxter is not of Cleveland's politics;—and competency, seniority of rank, long and efficient service—all go for naught if the applicant is not of the party having the "ins," —"to the victor belongs the spoils," evidently, is a cardinal maxim in politics.

### DEATH OF DR. JOSIAH T. MATTHIS, OF AUSTIN.

This estimable gentleman and skilled physician died November 30th, after an illness of only two weeks, of an obscure disease of the liver, the nature of which was only revealed to the physicians on post mortem examination. Dr. Mathis came to Texas from Tennessee some twelve years ago, settling in the southeastern part of the state, whence he removed to Austin two years later. Devoting himself exclusively to diseases of the eye, etc., he soon had a lucrative practice and made for himself a wide reputation as an oculist. He was a most estimable gentleman and an exemplary citizen, and his loss will be severly felt in this community, where he was universally respected. Aged 59 years. The Travis County Medical Association attended the funeral in a body.

